# Toward the Detection Limit of Electrochemistry: Studying Anodic Processes with a Fluorogenic Reporting Reaction

**DOI:** 10.1021/acs.analchem.3c00694

**Published:** 2023-07-17

**Authors:** Steven Linfield, Sylwester Gawinkowski, Wojciech Nogala

**Affiliations:** Institute of Physical Chemistry, Polish Academy of Sciences, Kasprzaka 44/52, 01-224 Warsaw, Poland

## Abstract

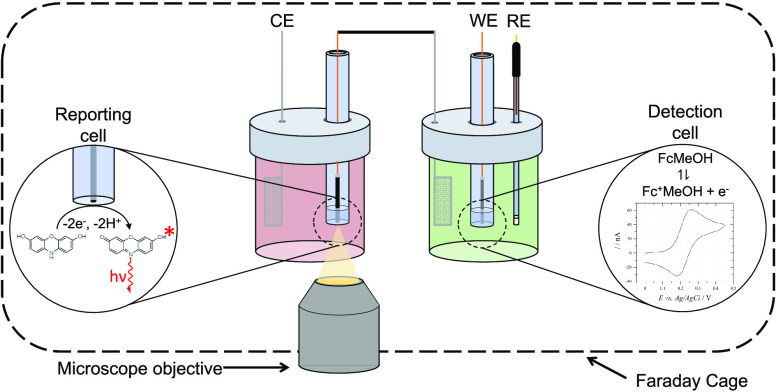

Recently, shot noise has been shown to be an inherent part of all charge-transfer processes, leading to a practical limit of quantification of 2100 electrons (≈0.34 fC) [Curr. Opin. Electrochem.2020, 22, 170−177]. Attainable limits of quantification are made much larger by greater background currents and insufficient instrumentation, which restricts progress in sensing and single-entity applications. This limitation can be overcome by converting electrochemical charges into photons, which can be detected with much greater sensitivity, even down to a single-photon level. In this work, we demonstrate the use of fluorescence, induced through a closed bipolar setup, to monitor charge-transfer processes below the detection limit of electrochemical workstations. During this process, the oxidation of ferrocenemethanol (FcMeOH) in one cell is used to concurrently drive the oxidation of Amplex Red (AR), a fluorogenic redox molecule, in another cell. The spectroelectrochemistry of AR is investigated and new insights on the commonplace practice of using deprotonated glucose to limit AR photooxidation are presented. The closed bipolar setup is used to produce fluorescence signals corresponding to the steady-state voltammetry of FcMeOH on a microelectrode. Chronopotentiometry is then used to show a linear relationship between the charge passed through FcMeOH oxidation and the integrated AR fluorescence signal. The sensitivity of the measurements obtained at different timescales varies between 2200 and 500 electrons per detected photon. The electrochemical detection limit is approached using a diluted FcMeOH solution in which no faradaic current signal is observed. Nevertheless, a fluorescence signal corresponding to FcMeOH oxidation is still seen, and the detection of charges down to 300 fC is demonstrated.

The boundaries of what can be measured electrochemically have been advanced by recent progress in nanoconfinement techniques,^1−6^ amplification strategies,^7−9^ and electrochemical instrumentation.^10,11^ Such developments have made it possible to measure electrochemical processes involving small amounts of charge being passed over short periods of time, down to currents on the order of femtoamps. However, the White group recently demonstrated that the inherent stochastic motion of electrons (*i.e.*, shot noise) produces a practical electrochemical limit of quantification of 2100 electrons, which corresponds to a charge of ≈0.34 fC.^12^ Furthermore, the presence of background currents and the shortcomings of instrumentation in experimental electrochemistry push this limit of quantification to higher values. As a result, several literature examples of low-current electrochemistry were shown to have come very close to the limit of quantification, highlighting the need to find ways of boosting the sensitivity of electron counting for future progress in fields such as nanoelectrochemistry and single-entity electrochemistry.^13^ One possible method of overcoming the detection limit is to employ the enhanced sensitivity of photon detection, which can be performed down to single-molecule and single-photon levels,^14−16^ by using an electrochemically induced luminescence signal to report on charge-transfer processes. While conventional means of improving the sensitivity of electrochemical measurements (*i.e.*, improving the electronics or utilizing amplification techniques) have limits imposed by the shot noise, the coupling of electrochemistry to luminescence avoids these limits due to the increased sensitivity of photon detection. Such high-sensitivity photon measurements have already been linked to electrochemically generated fluorescence^17^ and electrochemiluminescence.^18^

An expanding number of molecules have been reported to have luminescent properties directly related to their redox state.^19−21^ Coupling the luminescence of these molecules to the electrochemistry of interest, either directly or indirectly, is becoming increasingly popular for visualization of electrode processes.^22−24^ Luminescence induced by direct electron transfer can reveal the heterogeneity of an electrode and allow the activity of the electrode to be spatially resolved in situations where current can only give an averaged view of activity. An example of a fluorophore used to directly couple to electrochemistry is resorufin, a molecule which can be generated by the oxidation of the nonfluorescent dihydroresorufin molecule (or its nonfluorescent derivative Amplex Red (AR), see Scheme 1) or by the reduction of the less fluorescent resazurin molecule.^25^ The electrochemical generation of this molecule has been studied using confocal fluorescence microscopy, providing information about its reaction mechanism and the thickness of its diffusion layer.^26,27^ In another example, the 5-cyano-2,3-di(*p*-tolyl)tetra-zolium chloride fluorophore was irreversibly deposited into the gaps of an insulating TiO_2_ layer on top of a microelectrode, revealing fluorescent islands that were used to map out the defect sites of the TiO_2_ layer.^28^ In a demonstration of increased sensitivity and temporal resolution, the fluorescence of Nile red was recently used to detect the impact of attoliter droplets of toluene emulsions on a carbon fiber electrode.^29^ The electrochemiluminescence of tris(bipyridine) ruthenium(II) ([Ru(bpy)_3_]^2+^) has also been used to indicate the distribution of potential in a bipolar electrochemical cell and observe the real-time passivation of silicon surfaces.^30^ Luminescence induced by indirect electron transfer can reveal changes in local stimuli and uncover the kinetics of the electron transfer. Confocal fluorescence microscopy of the pH-sensitive fluorescein molecule has been used for 3-dimensional mapping of the proton gradient at a microelectrode and for evaluation of the number of electrons passed during the oxygen reduction reaction^31,32^ Another pH-sensitive fluorophore, 8-hydroxypyrene-1,3,6-trisulfonic acid (HPTS), has been used to study the reaction layer of p-benzoquinone reduction around a carbon fiber inside a thin-layer cell.^33^ Recently, the concept of electrochemically induced luminescence has been applied to remotely report on electrochemistry occurring in a separate cell.

**Scheme 1:**
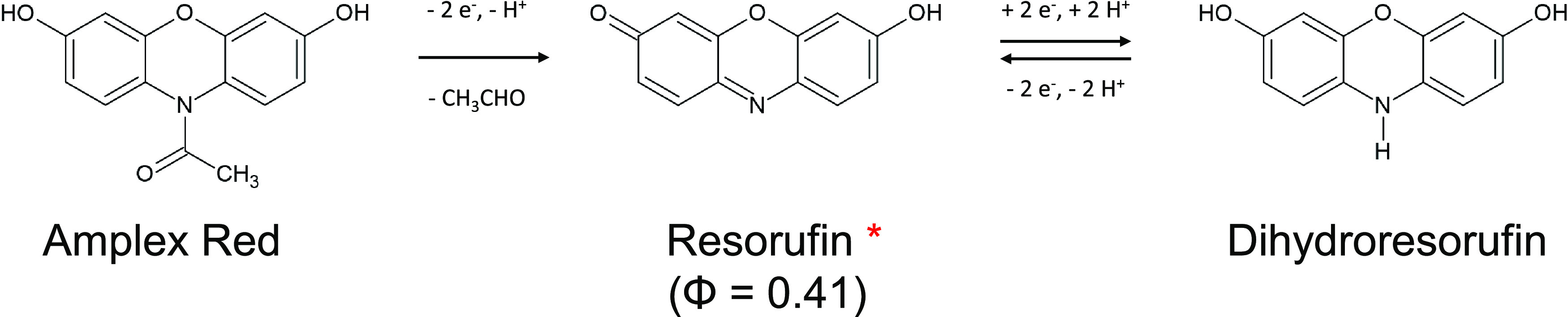
Mechanism for Amplex Red Oxidation The Φ symbol denotes the quantum yield of the resorufin molecule.

The ability to induce luminescence in a separate cell allows any electrochemical process to be reported on, not just those which can directly or indirectly change the redox state of a local luminophore. The first example of remote reporting used a closed bipolar microelectrode array to monitor the oxidation of ferrocenemethanol in one cell, referred to as the detection cell, through the change in quantum efficiency that accompanied the reduction of resazurin to resorufin in another cell, referred to as the reporting cell.^34^ This was used to image the heterogeneity of a Pt catalyst deposited on the detection cell side of the array, but could only be used to report on oxidation reactions. Continued work led to the same system being used to report on the reduction of hexacyanoferrate(III) through the oxidation of dihydroresorufin to resorufin.^35^ This method of remote reporting has since been used to detect μM concentrations of hydrogen peroxide and glucose,^36^ map the diffusion field of hexacyanoferrate at a microelectrode,^37^ and report on the hydrogen evolution occurring at a Pt nanoparticle.^38^ Similar systems using the electrochemiluminescence of [Ru(bpy)_3_]^2+^ were shown to successfully report the reduction of ferricyanide and detect the impact of Pt nanoparticles on a microelectrode.^39,40^ Another type of remote reporting was recently demonstrated using a conventional three-electrode system, with the working and reference electrodes in the detection cell separated from the counter electrode in the reporting cell with a glass frit.^41,42^ The advantage of this cell design over the traditional closed bipolar system was that the three-electrode cell allowed for direct thermodynamic control over the electrochemistry of interest. Electrochemical signals of nanoamps were successfully reported through fluorescence induced by the passage of current at the counter electrode. However, this system still required different fluorogenic reactions depending on whether oxidation or reduction was performed in the detection cell. The reduction of p-benzoquinone in the presence of HPTS in the reporting cell was used to report oxidation of ferrocenedimethanol in the detection cell, while the oxidation of AR to resorufin in the reporting cell was used to report on the reduction of hexaammineruthenium(III) in the detection cell. In principle, any electroactive fluorophore can be used to remotely report on electrochemistry in a separate cell, provided it meets a few conditions. The oxidized and reduced form of the fluorophore must both be stable enough to be measured electrochemically and their emissions must be distinguishable from each other, either through a change in the quantum yield or through a shift in the emission wavelength.

To our knowledge, there have been no attempts to use a remote reporting reaction to circumnavigate the electrochemical detection limit or to develop a system in which the same luminophore can be used regardless of whether oxidation or reduction is performed in the detection cell. Herein, we demonstrate the use of a remote fluorogenic reporting reaction to measure charges close to the electrochemical limit of quantification and we compare the efficiencies of the reporting reaction under different experimental timescales. This is a large step toward the quantitative conversion of subdetection limit electrochemical signals into more sensitive photon signals. This advancement will improve the sensitivity of single-entity electrochemistry and sensing applications which use remote optical reporting, allowing smaller entities and lower concentrations of analyte to be detected.^40,43^ We also present an alternative system for remote reporting in which fluorescence is induced through microelectrodes in a closed bipolar electrochemical cell in three-electrode driving mode^44^ and measured using a conventional epifluorescence microscope. This allows for direct thermodynamic control of the electrochemistry of interest, while enabling the same fluorogenic reaction, the oxidation of AR, to be used to report on either reduction or oxidation processes. We also discuss the commonplace practice of chemical quenching of fluorophores in remote reporting systems and its implications on the performance of the reporting cell.

## Experimental Details

### Chemicals and Solutions

Amplex Red (AR) was purchased from Thermo Fisher Scientific Inc. Ferrocenemethanol (FcMeOH; 99%) was purchased from abcr GmbH. Dimethylsulfoxide (DMSO; p.f.a.), d-(+)-glucose (C_6_H_12_O_6_; p.f.a.), and potassium sulfate (K_2_SO_4_; p.f.a) were purchased from Chempur. Sodium hydroxide (NaOH; p.f.a.) was purchased from Avantor Performance Materials Poland S.A. Isopropanol ((CH_3_)_2_CHOH; p.f.a) was purchased from PPH STANLAB Sp. z o. o. Unless stated otherwise, all solutions were prepared using deionized water (18.2 MΩ cm) from an Arium Comfort water purifier from Sartorius AG.

### Solutions and Cell Setup

A stock solution (10 mM) of AR was prepared by dissolving the AR (5 mg) in DMSO (1.944 mL). An aliquot of this stock solution was added to the reporting cell, along with a solution of C_6_H_12_O_6_ (0.1 M) in NaOH (0.5 M). The volumes of each solution varied depending on the desired concentration, but the total volume was always 1 mL. Around 1 mL of the FcMeOH solution (concentrations varied) was added to the detection cell. The design and dimensions of the electrochemical cell can be found in Figure S1.

### Electrochemical Setup

Carbon fiber (CF) microelectrodes (7 μm ⌀ and 33 μm ⌀) and a silver/silver chloride (Ag/AgCl) electrode were purchased from ALS Co., Ltd. Platinum (Pt) microwire (500 μm) was purchased from Carl Roth GmbH. These electrodes were arranged in the detection and reporting cells according to Table 1.

**Table 1:** Placement of the Working Electrode (WE), Counter Electrode (CE), Reference Electrode (RE), and the Two Poles of the Closed Bipolar Electrodes (BPE1 and BPE2)^a^1

detection cell	
WE	7 μm ⌀ CF electrode
BPE1	500 μm ⌀ Pt wire
RE	Ag/AgCl

reporting cell	
CE	500 μm ⌀ Pt wire
BPE2	33 μm ⌀ CF electrode

a Setup details can be seen in Figure 1.

Note that in this setup, a micropositioner was used to place BPE2 at a distance of 500 μm from the microscope slide. A PalmSens4 potentiostat from PalmSens B.V. was used for potentiostatic and galvanostatic control of the working electrode. Electrochemical measurements were recorded through PSTrace 5.9 software. The potential of the reference electrode was checked against a saturated calomel electrode, and any potential shifts were accounted for on potentiostat settings.

An illustration of the electrochemical setup is shown in Figure 1. Electromagnetic noise was reduced by performing measurements inside a Faraday cage. This consisted of two parts: a copper lid and a copper plate with a hole for a microscope objective, allowing observation of the fluorescence in the reporting cell, and a small opening for the potentiostat cables. Remaining noise picked up by an unshielded connecting wire between the bipolar electrodes could also influence the electrochemical measurements, so a shielded coaxial cable was used with its shield connected to the Faraday cage.

**Figure 1:**
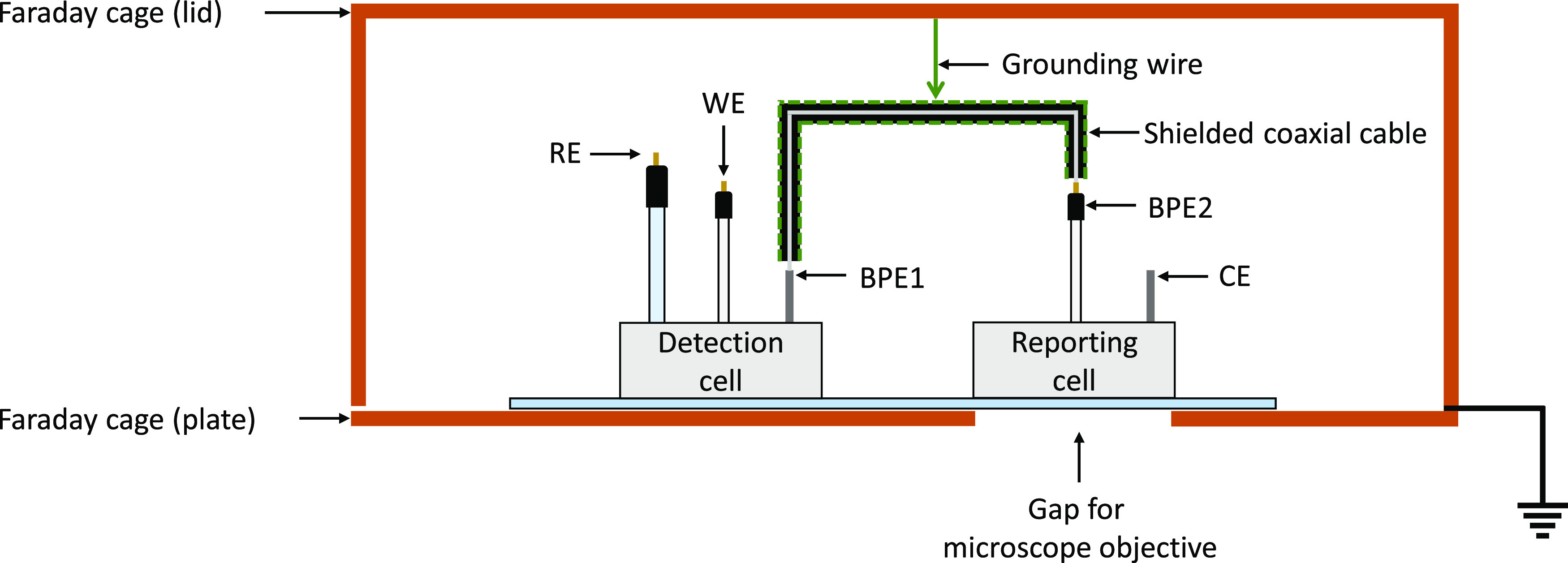
Diagram showing the arrangement of electrodes in the closed bipolar electrochemical cell.

**Figure 2:**
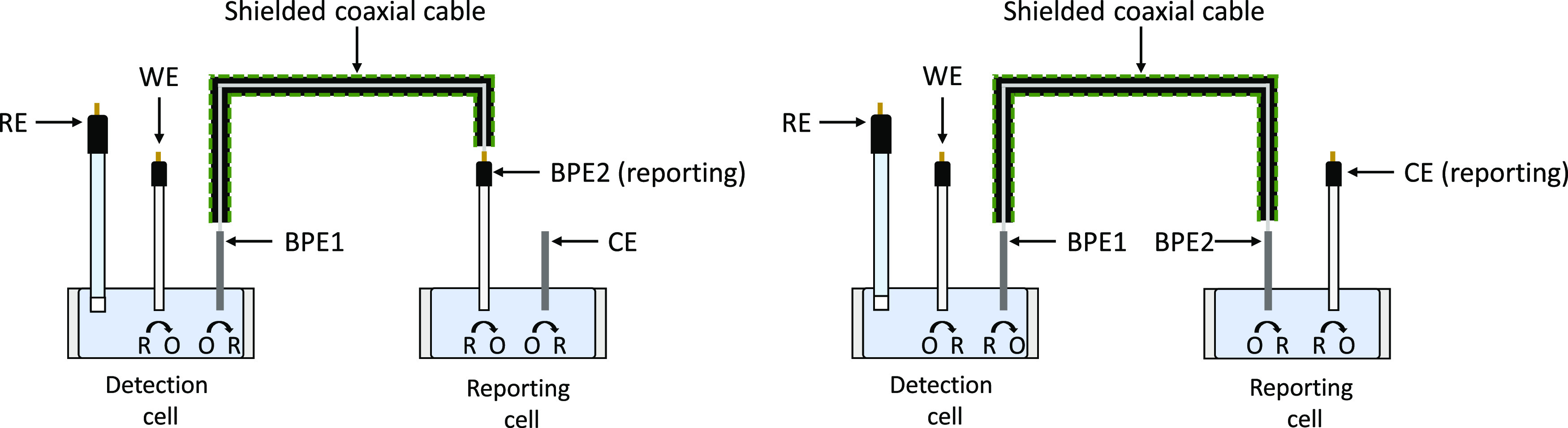
Diagram showing how the polarity of the reporting electrode is related to the polarity of the working electrode and how the position of the carbon fiber electrode in the reporting cell can allow the same fluorophore to be used to report of both anodic and cathodic reactions.

One of the advantages of the three-electrode driving mode closed bipolar electrochemical setup is that it allows the position of the carbon fiber electrode within the reporting cell to be switched between being the second pole of the bipolar electrode (BPE2) and being the counter electrode (CE). As shown in Figure 2, this allows the same fluorophore to be used to report on both anodic and cathodic processes, since electrodes in the working electrode (WE) and the BPE2 positions share the same polarity, while electrodes in the WE and CE positions have the opposite polarity.

### Measurement of Fluorescence Signal

The electrochemical setup was placed on the stage of an Eclipse Ti2 optical microscope from Nikon Corporation. This microscope was connected to an ORCA-Flash4.0 V3 digital complementary metal oxide semiconductor (CMOS) camera from Hamamatsu Photonics K.K., which was controlled using Nikon Elements software.

The nosepiece of the optical microscope was equipped with 2.5× (numerical aperture (NA) = 0.075), 10× (NA = 0.3), 20× (NA = 0.5), and 60× (NA = 0.7) objectives. The stage was moved with a manual XY positioner until the carbon fiber disc was in the center of the brightfield image. The image of the CF was then focussed by moving the height of the nosepiece with a ES10ZE/D motor controller from Prior Scientific Instruments Ltd. This process was repeated on every objective (from lower to higher magnification) until reaching the objective which was to be used. Unless specified, all measurements herein were recorded using the 60× objective. During this process, the electrode was illuminated using light (intensity varied) produced by a pE-300lite LED purchased from CoolLED Ltd. During focusing, the exposure time and lookup tables (LUTs) of the camera were set to automatic. When the image was in focus, a region of interest (ROI) was drawn around the carbon disc.

When measuring fluorescence, the LED light was passed through a filter cube fitted with a TRITC-A filter set (λ_excitation_ = 543–566 nm, λ_emission_ = 582–636 nm) from Semrock Inc. This allowed emission of the resorufin (peak λ_excitation_ = 571 nm, peak λ_emission_ = 585 nm, see Figure S2) to be separated from the excitation. The exposure time was set to a value between 50 and 200 ms, and LUTs were adjusted to accommodate the maximum pixel intensity observed during fluorescence. A rainbow false color map was used to better visualize the fluorescence. The sum of pixel intensities in the ROI was measured over time and saved in .txt files. The background intensity was subtracted from each measurement, allowing the change in the sum of intensities to be compared and displayed in the figures seen herein. The Supporting Information contains additional information on how the integration of the fluorescence signal was performed for certain figures shown herein. The image of the electrode during the fluorescence was saved as a .mp4 file.

## Results and Discussion

### Electrochemistry of the Reporting Cell

To report on electrochemistry measured in the detection cell, we need an understanding of the processes which may occur in the reporting cell and how the design of the closed bipolar cell can influence the reporting process. For example, the most commonly used fluorogenic redox molecule to report electrochemistry, Amplex Red (AR), is extremely susceptible to photooxidation.^45^ In this process, the fluorescent resorufin is continually produced in any environment with sufficient oxygen and illumination. The accumulation of resorufin results in an increasing background fluorescence during the course of an experiment (Video S0). Furthermore, the rate of photooxidation appears to be greater in solutions that have already undergone some photooxidation (Figures S3 and S4). To limit the impact of photooxidation, some literature sources suggest replacing conventional electrolytes with glucose and sodium hydroxide.^35,36^ Deprotonated glucose, which is generated in the presence of sodium hydroxide, reportedly acts as a chemical reductant, reducing any resorufin produced during photooxidation to dihydroresorufin. Glucose has a p*K*_a_ of ≈12.5, so a NaOH concentration of 0.5 M was selected to ensure glucose could be deprotonated.^46^ There has been some optimization of the glucose concentrations in the literature suggesting that concentrations between 67 and 250 mM do not diminish the strength of fluorescence induced by electron transfer.

**Figure 3:**
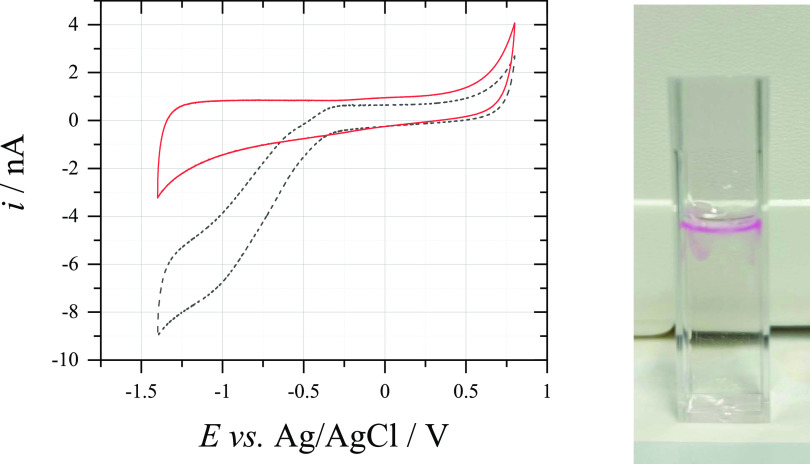
(Left) Cyclic voltammogram (500 mV s^–1^) recorded at a 33 μm ⌀ CF electrode in aerobic solutions of (black, dashed) 0.5 M NaOH and (red, solid) 0.1 M C_6_H_12_O_6_ + 0.5 M NaOH. (Right) Cuvette filled with an aerobic solution of 10 μM Amplex Red + 0.1 M C_6_H_12_O_6_ + 0.5 M NaOH.

The introduction of glucose imposes some restrictions on the electrode material which can be used in the reporting cell. Platinum and gold can promote significant background glucose electrochemistry (Figure S5) which may detract from the charge passed to induce AR oxidation.^47^ To avoid this, carbon electrodes are used in the reporting cell, since carbon shows no activity toward glucose oxidation. The voltammogram in Figure 3 shows the different responses of a carbon fiber (CF) microelectrode in aerobic sodium hydroxide before and after the addition of glucose. While no glucose oxidation is observed, the oxygen reduction reaction wave starting at ≈−0.3 V *vs* Ag/AgCl is no longer present. This suggests that deprotonated glucose is capable of scrubbing oxygen from the solution, providing an additional mechanism by which it can counter photooxidation. The solution of AR in glucose and sodium hydroxide shown in Figure 3 only exhibits signs of photooxidation (*i.e.*, the pink resorufin color) near the liquid–air interface. The pH of the NaOH exceeds the p*K*_a_ of AR (8.5) and since the oxidation of AR requires loss of a proton (and the oxidation of dihyrdoresorufin requires loss of two protons), Le Chatelier’s principle predicts that significant photooxidation would be expected throughout the whole solution at the pH values used. Instead, the rest of the solution is clear, which is consistent with the combined mechanism of chemical reduction and oxygen scrubbing by glucose.

**Figure 4:**
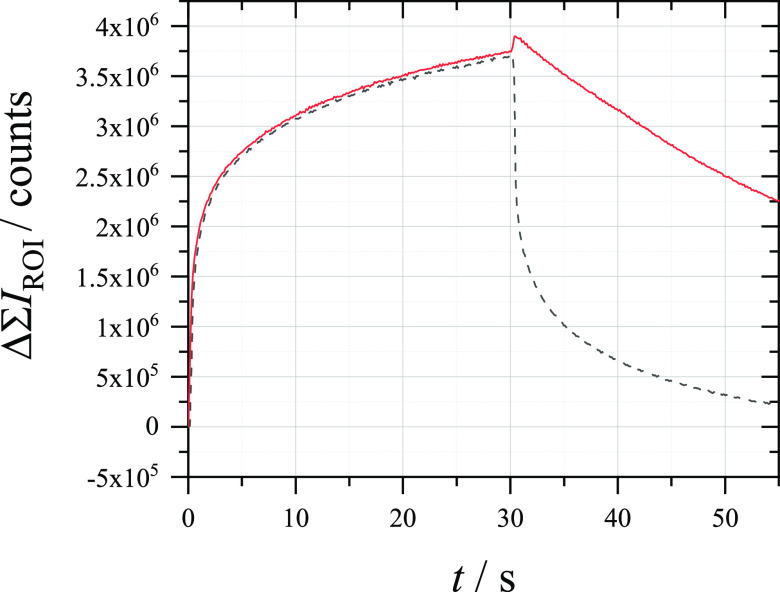
Change in sum fluorescence intensity measured at a 33 μm ⌀ CF electrode (reporting) in an aerobic solution of 10 μM Amplex Red + 0.1 M C_6_H_12_O_6_ + 0.5 M NaOH during chronoamperometry (from 0 to +0.5 V *vs* Ag/AgCl for 30 s) measured at a 7 μm ⌀ CF electrode (detection) in an aerobic solution of 50 μM FcMeOH + 0.1 M K_2_SO_4_. After the experiment, the cell was either kept on at 0 V *vs* Ag/AgCl (black, dashed) or switched to OCP (red, solid).

It should also be noted that if glucose reduces resorufin produced by photooxidation, it should also reduce resorufin produced by charge passed through the closed bipolar electrochemical cell (*i.e.*, an EC′ mechanism with a pseudo first-order rate constant, since glucose is in excess). It has been suggested that this may allow for a direct relationship between the current and the fluorescence signal, as opposed to needing to integrate the fluorescence signal over time.^35−37^ However, the fluorescence response to chronoamperometry induced through a closed bipolar cell, shown in Figure 4, indicates that the rate constant of the chemical step is not fast enough for a direct relationship between the current and the fluorescence signal. When the working electrode is later held at a reduction potential for the generated hydroxymethyferrocenium cations in the detection cell, the fluorescence observed in the reporting cell dropped exponentially. When the electrode was held at open circuit potential (OCP) after the chronoamperometry, the decline in the fluorescence signal was much slower. This implies that homogeneous chemical reduction by deprotonated glucose is less efficient than electrochemical reduction and that integration of the fluorescence signal is still needed to extract information about the electrons passed (at least under the conditions used in Figure 4; other conditions, such as the use of smaller electrodes, may allow for a direct relationship to be used). Moreover, the galvanostatic OCP mode experiment with the employed potentiostat was shown to be affected by a leakage current corresponding to higher current densities at the reporting microelectrode (Figure S6). This produces a small increase in the fluorescence signal after switching to OCP.

**Figure 5:**
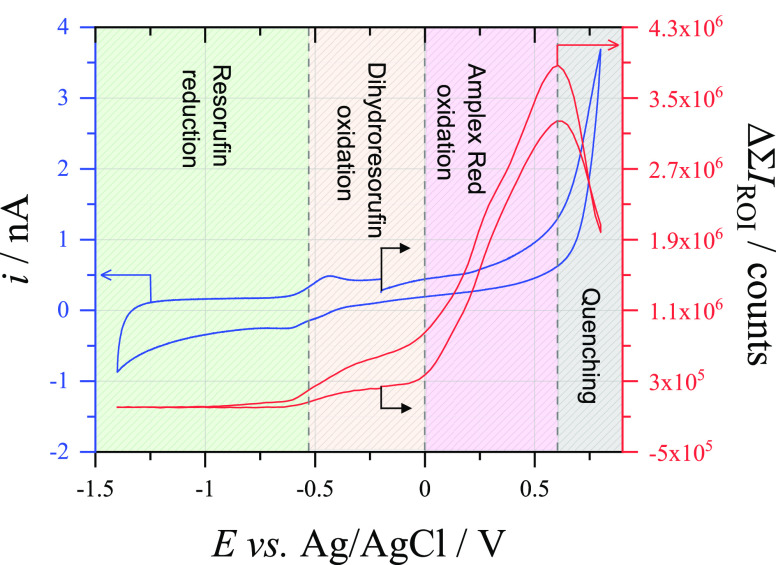
(Blue) Cyclic voltammogram (100 mV s^–1^) recorded at a 33 μm ⌀ CF electrode in an aerobic solution of 0.1 mM Amplex Red + 0.1 M C_6_H_12_O_6_ + 0.5 M NaOH and (red) the change in sum fluorescence intensity measured through a 20× microscope objective at a 33 μm ⌀ CF electrode during cyclic voltammetry (100 mV s^–1^) in an aerobic solution of 10 μM Amplex Red + 0.1 M C_6_H_12_O_6_ + 0.5 M NaOH. The black arrows indicate the position and direction of the scan on each plot.

Reduction by glucose should also cause the voltammetry of AR to take the appearance of that with an EC′ mechanism, in which the electrochemical reduction of resorufin would not be seen at slow scan rates. However, the voltammetry observed in Figure 5 does not seem to reflect this, with reduction/oxidation peaks for the resorufin–dihydroresorufin couple visible around −0.5 V *vs* Ag/AgCl, alongside a wave corresponding to AR oxidation starting around 0 V *vs* Ag/AgCl.^27^ Once more, this implies the rate of chemical reduction of resorufin is too slow to allow for direct relationship between the current and the fluorescence signal and that integration of the fluorescence signal is still needed to quantify the amount of charge that has been passed through the reporting electrode. More work is needed to fully understand how the efficiency of the chemical reduction step is dependent on factors such as the concentration of glucose and the electrode size, but is beyond the scope of this work.

It is also important to understand the relationship between the current passed at the reporting electrode and the fluorescence signal. As seen in Figure 5, the fluorescence signal correlates well to the voltammetric signal, increasing dramatically between 0 and +0.6 V *vs* Ag/AgCl as AR is oxidized and later decreasing to background levels as the fluorescent resorufin is reduced to nonfluorescent dihydroresorufin both chemically and electrochemically. However, it can also be seen that above +0.6 V *vs* Ag/AgCl the fluorescence signal sharply drops, while at the same potential there is a rise in current. Clearly, some process above this potential is quenching the fluorescence, most likely due to further oxidation of the resorufin compound to resazurin.^48^ The quenching process needs to be avoided when the reporting electrode is held under galvanostatic control or applied in the bipolar setup for conversion of faradaic current to fluorescence. Therefore, the current passed in the detection cell must not exceed the limiting current of AR oxidation in the reporting cell.^49^ This could be achieved by using either a larger electrode in the reporting cell or an increased AR concentration to make sure that its oxidation is not the limiting process. In reality, these measures are limited by competing reactions at AR concentrations higher than 50 μM and by excessive charge consumption during the double-layer charging of a large reporting electrode.^50^ Hence, to avoid the loss of faradaic charge toward charging of the electrical double layer, it is practical to use electrodes that are similar in size to compensate for charging currents and to ensure that the currents measured in the detection cell do not breach an upper threshold.

### Remote Reporting of Electrochemistry on a Microelectrode

Microelectrodes are convenient for the investigation of electrochemistry near the limit of quantification in the detection cell, due to their reduced capacitance and their ability to establish a steady-state diffusion field. Furthermore, their small dimensions enable the use of higher-magnification objectives, which tend to have higher numerical apertures which can collect more light (Figures S7 and S8), thereby increasing the measured signal at the reporting cell. The expected response when using microelectrodes in the remote reporting system can be investigated by monitoring the oxidation of ferrocenemethanol (FcMeOH) in the detection cell through electrogenerated fluorescence of resorufin in the reporting cell.

**Figure 6:**
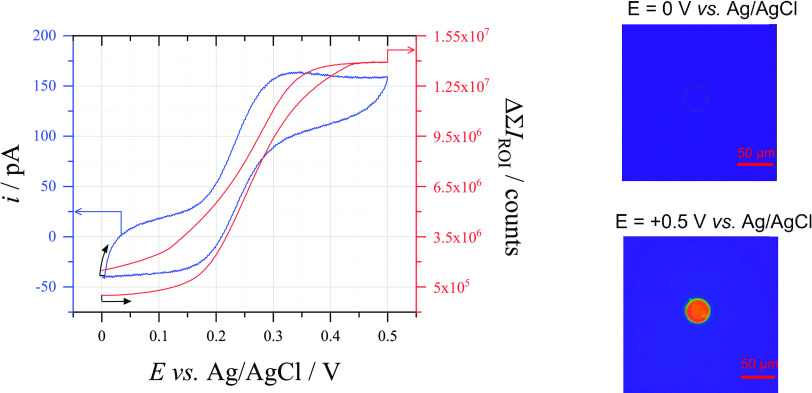
(Left) (blue) Cyclic voltammogram (25 mV s^–1^) recorded at a 7 μm ⌀ CF electrode (detection) in an aerobic solution of 0.25 mM FcMeOH + 0.1 M K_2_SO_4_ and (red) the change in sum fluorescence intensity concurrently measured at a 33 μm ⌀ CF electrode (reporting) in an aerobic solution of 40 μM Amplex Red + 0.1 M C_6_H_12_O_6_ + 0.5 M NaOH. The black arrows indicate the position and direction of the scan on each plot. (Right) Microscopy images (taken from Video S1) of the 33 μm ⌀ CF electrode during voltammetry of the 7 μm ⌀ CF electrode (scale bar denotes 50 μm).

A sigmoidal voltammogram typical for the oxidation of FcMeOH on a microelectrode is seen in Figure 6. The capacitive current observed in the voltammetry is caused by the surface roughness of the carbon fiber microelectrode, but is still only around 30 pA in magnitude. Concurrent measurement of the fluorescence signal induced through the closed bipolar electrochemical cell also appears to follow a sigmoidal shape, due to the establishment of steady-state conditions at the reporting electrode. However, the fluorescence signal is shifted to more positive potentials compared to the electrochemical signal and its hysteresis is inverted. This indicates a delay between the passage of current through the cell and the onset of fluorescence, possibly due to the chemical reduction by glucose at low overpotentials. On the reverse scan, the working electrode returns to potentials where the oxidation of FcMeOH is no longer possible, resulting in both signals returning to baseline values. The fluorescence signal is delayed which indicates that the fluorophore lingers in the reporting cell after the current is passed. The specific selection of 33 μm ⌀ CF for the reporting electrode was influenced by the need to use the largest electrode that could fit in the field of view of the 60× microscope objective. The 7 μm ⌀ CF for the detection electrode was chosen to ensure that the current from FcMeOH oxidation did not exceed the current of Amplex Red oxidation in the reporting cell, which as mentioned before would induce the further oxidation of resorufin to resazurin.

**Figure 7:**
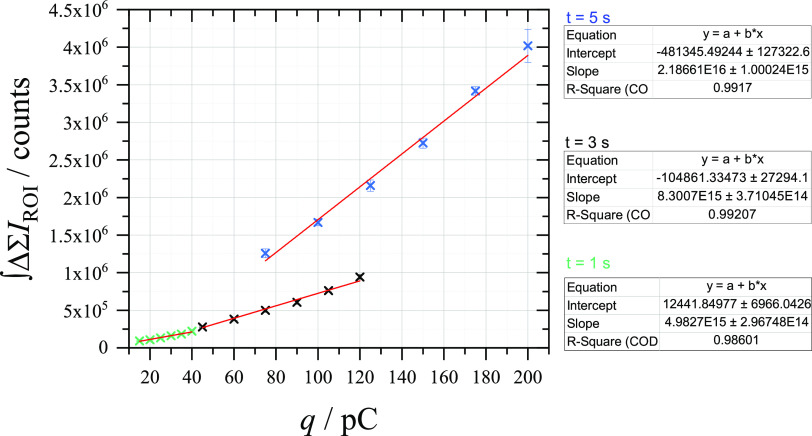
Relationship between the average (10×) integrated change in sum intensity recorded at a 33 μm ⌀ CF electrode (reporting) in an aerobic solution of 10 μM Amplex Red + 0.1 M C_6_H_12_O_6_ + 0.5 M NaOH while performing chronopotentiometry (15–40 pA for (blue) 5 s, (black) 3 s, and (green) 1 s) at a 7 μm ⌀ CF electrode (detection) in an aerobic solution of 50 μM FcMeOH + 0.1 M K_2_SO_4_.

One can observe the influence of these temporal effects on the reporting reaction by passing known charges at the working electrode and comparing the fluorescence signals measured at the reporting electrode during various timescales. As shown in Figure 7, the fluorescence measured at each timescale shows a linear relationship with the amount of charge passed through the closed bipolar electrochemical cell. However, the gradient of this relationship appears to change with the timescale used, with longer passage of charge leading to larger fluorescence signals. The gradients at each timescale correlate to electrons photon^–1^ values of 2179 ± 138 for 1 s pulses, 1308 ± 61 for 3 s pulses, and 496 ± 24 for 5 s pulses. This is consistent with the observation that the fluorophore lingers near the reporting electrode after the charge has been passed and suggests that the issue is more prominent as time increases. The same phenomenon can be observed during a pulsed amperometric detection experiment (Figure S9), in which fluorescence is observed at longer timescales than the pulse length. The implication here is that the fluorescence produced on different timescales cannot be accurately compared. However, this also may make fluorescence a better candidate for reporting on subdetection limit electrochemistry, as the lingering fluorescence signal would be easier to detect.

### Approaching the Limit of Detection

The same setup can be used to report electrochemistry below the detection limit of an electrochemical workstation. In this work, this was demonstrated by repeating electrochemistry of FcMeOH in the detection cell, but with a more diluted solution. This approach decreases the faradaic current below the charging current, making it effectively invisible with cyclic voltammetry.

**Figure 8:**
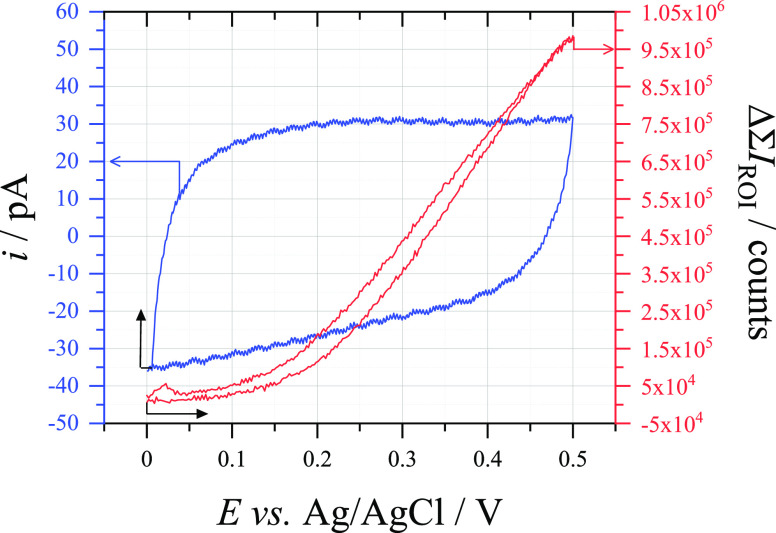
(Blue) Cyclic voltammogram (25 mV s^–1^) recorded at a 7 μm ⌀ CF electrode (detection) in an aerobic solution of 2.5 μM FcMeOH + 0.1 M K_2_SO_4_ and (red) the change in sum fluorescence intensity concurrently measured through a 20× objective at a 33 μm ⌀ CF electrode (reporting) in an aerobic solution of 10 μM Amplex Red + 0.1 M C_6_H_12_O_6_ + 0.5 M NaOH. The black arrows indicate the position and direction of the scan on each plot.

Voltammetry of the diluted FcMeOH solution, shown in Figure 8, gave no visible faradaic current. This is due to the expected limiting current for FcMeOH oxidation, which in these conditions was ≈2.5 pA, being smaller in magnitude than the capacitive current of the electrode. A linear increase in the fluorescence is observed during the voltammetry, which could be due to many different processes (both faradaic and nonfaradaic) including background faradaic processes of trace impurities, electrochemical noise, double-layer charging, and electron transfer from the diluted FcMeOH. The fluorescence signal observed in Figure 8 is a result of all of these processes. The linear appearance of the fluorescence signal here in comparison to the sigmoidal appearance of the signal in Figure 6 is due to the lower contribution from FcMeOH oxidation, making the double-layer charging contribution more prominent. However, to extract the precise contribution of the faradaic processes, it would be necessary to subtract a background intensity profile recorded in only the electrolyte (see Figures S10 and S11).

**Figure 9:**
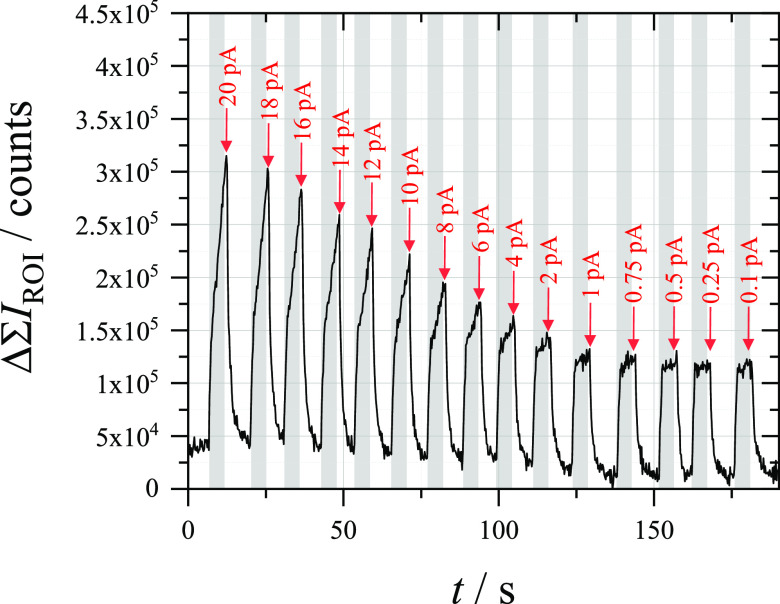
Change in sum fluorescence intensity measured at a 33 μm ⌀ CF electrode (reporting) in an aerobic solution of 10 μM Amplex Red + 0.1 M C_6_H_12_O_6_ + 0.5 M NaOH during continuous chronopotentiometry (at the indicated current levels for 5 s each) measured at a 7 μm ⌀ CF electrode (detection) in an aerobic solution of 2.5 μM FcMeOH + 0.1 M K_2_SO_4_. The gray areas indicate when chronopotentiometry was measured.

When closer to the detection limit, chronopotentiometry can be once again used to look at the relationship between charge passed in the detection cell and fluorescence observed in the reporting cell. As shown in Figure 9, the fluorescence signal decreases as the charge passed is reduced, with charges as low as 5 pC observed by changing the current flowing through the working electrode. Given an expected mass transport limited current of ≈2.5 pA, many of the charges passed here are fulfilled by evolution of oxygen in the detection cell. It should be noted that the exact value of the current applied to the working electrode is not known. According to the potentiostat’s specifications, the galvanostatic control has a stated accuracy of ±1% of the current range and an additional ±10 pA. While it is clear that the fluorescence decreases with applied current, for better accuracy, it would be necessary to use potentiostatic control on even smaller electrodes (*i.e.*, nanoelectrodes) or an analogue galvanostat composed of a stable voltage source and a resistor of much larger resistance than the impedance of the studied cell in series. Currents below 1 pA were not applicable using the galvanostatic mode, resulting in each signal below this current having the same fluorescence signal. In order to see if smaller charges could be observed, the timescale of the chronopotentiometry could also be reduced.

**Figure 10:**
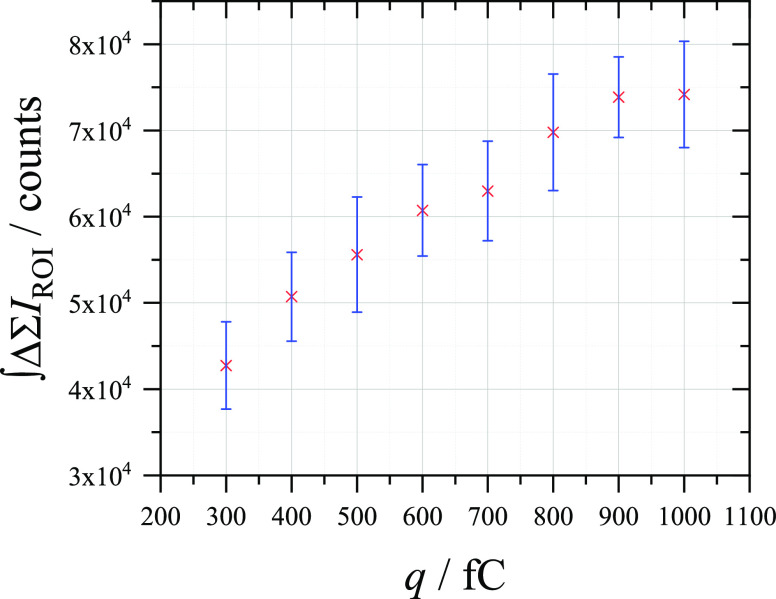
Relationship between the average (10x) integrated change in sum intensity recorded at a 33 μm ⌀ CF electrode (reporting) in an aerobic solution of 10 μM Amplex Red + 0.1 M C_6_H_12_O_6_ + 0.5 M NaOH while performing chronopotentiometry (1 pA for 0.3–1 s) at a 7 μm ⌀ CF electrode (detection) in an aerobic solution of 2.5 μM FcMeOH + 0.1 M K_2_SO_4_.

Figure 10 shows the change in the fluorescence intensity measured in the reporting cell, while changing the time for which the working electrode was held at 1 pA. This resulted in the passage of charges in the fC range and the measurement of fluorescence signals which appear to correspond to those charges, decreasing as the charge passed is decreased. Note that the chronopotentiometry for each charge was performed at a different timescale, and so no linear relationship as seen in Figure 7 can be assumed here. Despite averaging of the fluorescence signals, there was a wide error due to the timescale of the chronopotentiometry approaching the shortest exposure time of the CMOS camera (in this case, 200 ms). Further improvements in the photon detection and charge application are expected to improve the sensitivity of remotely reported charges to below the fC range shown here.

## Conclusions

Fluorescence induced through a closed bipolar electrochemical cell was used to demonstrate the measurement of electrochemistry approaching the detection limit. The use of microelectrodes in both the reporting and detection cell allowed for currents close to the limit of quantification to be observed without significant capacitive current, as well as enabling the use of objectives with a higher numerical aperture. Measurement of the integrated fluorescence signal produced by passage of charge showed a linear relationship which was also dependent on the timescale of the experiment. The processes in the reporting cell were also studied, and new insights on the use of glucose in countering photooxidation were proposed. There are still many improvements to be made to the reporting system shown here. Resolving the fluorescence signals observed at different timescales is crucial to calculating the charge that has passed, and better time-resolved signals could be achieved with alternative techniques like electrochemiluminescence. Potentiostatic control of nanoelectrodes and improvements to the sensitivity of photon detection will also help toward the conversion of charges below femtocoulombs. However, the data shown herein suggests that using a fluorogenic reaction to report electrochemistry below the detection limit is possible and is a key step toward next-generation nanoelectrochemistry.

## Supporting Information Available

Design of the closed bipolar electrochemical cell, absorbance and emission spectra of resorufin, influence and rate of photooxidation, background electrochemistry of glucose on Pt and Au electrodes, evidence for the passage of current when switching to OCP, maximizing the collection of fluorescence, example calculation of the gradients from Figure 7 and the integrated signal from Figures 7 and 10, further evidence for the lingering response of the resorufin, and fluorescence signal in K_2_SO_4_ with and without FcMeOH (PDF)
Video showing the increasing fluorescence background caused by photooxidation of Amplex Red/dihydroresorufin (Video S0) (MP4)
Video showing the fluorescence at the reporting electrode during voltammetry shown in Figure 5 (Video S1) (MP4)
