# Supplementary material for: Toward the Detection Limit of Electrochemistry: Studying Anodic Processes with a Fluorogenic Reporting Reaction
Source: Anal Chem. 2023 Jul 17;95(30):11227–35. doi: 10.1021/acs.analchem.3c00694 (PMC10398625; doi:10.1021/acs.analchem.3c00694)
Supplement: Supplementary file 1 — ac3c00694_si_001.pdf [file ac3c00694_si_001.pdf]

Supporting Information

Towards the detection limit of electrochemistry: Studying anodic processes with a fluorogenic reporting reaction

Steven Linfield,* Sylwester Gawinkowski, and Wojciech Nogala*

Institute of Physical Chemistry, Polish Academy of Sciences, Kasprzaka 44/52, 01-224 Warsaw, Poland

E-mail: slinfield@ichf.edu.pl; wnogala@ichf.edu.pl

Table of contents

Design of the closed bipolar electrochemical cell S2

Absorbance and emission spectra of resorufin S3

Influence and rate of photooxidation S4

Background electrochemistry of glucose on Pt and Au electrodes S6

Evidence for the passage of current when switching to OCP S7

Maximising the collection of fluoresced light S8

Example calculation of the gradients in Figure 7 . . . . S10

Further evidence for the temporal response of the fluorogenic reporting reaction . S12

Fluorescence signal in K_2SO_4 with and without FcMeOH S13

Example calculation of the integrated signal from Figures 7 and 10 . . . . S15

Design of the closed bipolar electrochemical cell

Custom electrochemical cells for closed bipolar electrochemistry were prepared by bonding two separate molded poly(dimethylsiloxane) (PDMS) structures to a glass microscope slide (76 mm × 26 mm × 1 mm) from Carl Roth GmbH.

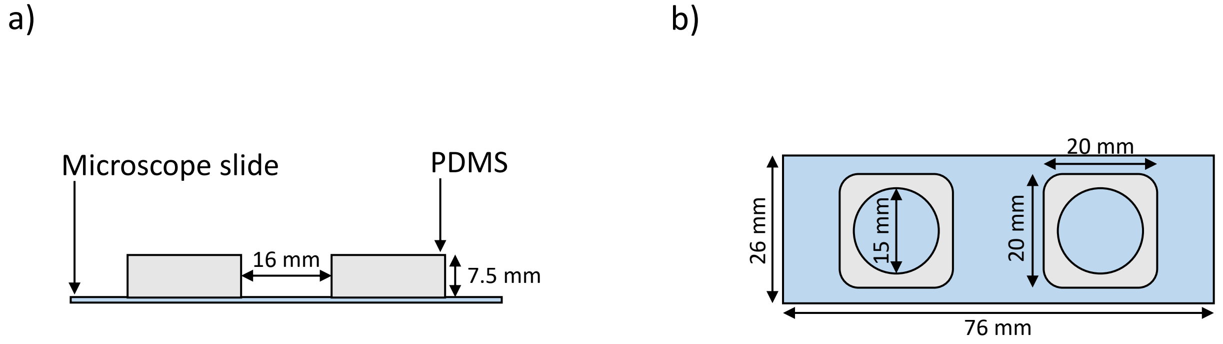

Figure S1: Diagram showing the dimensions and appearance of the closed bipolar electrochemical cell as viewed from (a) the side and (b) above.

Figure S1 illustrates the design, with two cells bound to each glass slide (one cell for detection and one for reporting). The dimensions of the templates resulted in each cell having a volume of ≈ 1.33mL. The molded PDMS structures were prepared using a Sylgard™ 184 Silicone Elastomer kit from Dow Europe GmbH. A 10:1 ratio of elastomer to curing agent (by weight) was vigorously mixed (2-5 minutes) before removal of gas bubbles under vacuum (20-30 minutes). The mixture was then poured into a Teflon mold, covered with a glass slide (to provide a smooth upper surface), and left to set in a 75°C oven overnight. The PDMS cell was then removed from the Teflon mold, washed with isopropanol, and left to dry.

To bond the PDMS to the microscope slide, both the slide and the PDMS structure (smooth side) were cleaned with isopropanol, dried, and placed face up on a sheet of aluminium foil in the chamber of a Harrick Plasma pdc-002-ce plasma cleaner. The surfaces were exposed to plasma for one minute to generate free radicals, then quickly placed on top of each other. To finish, the foil with the microscope slide was transferred to a 125°C hotplate (15 minutes).

Absorbance and emission spectra of resorufin

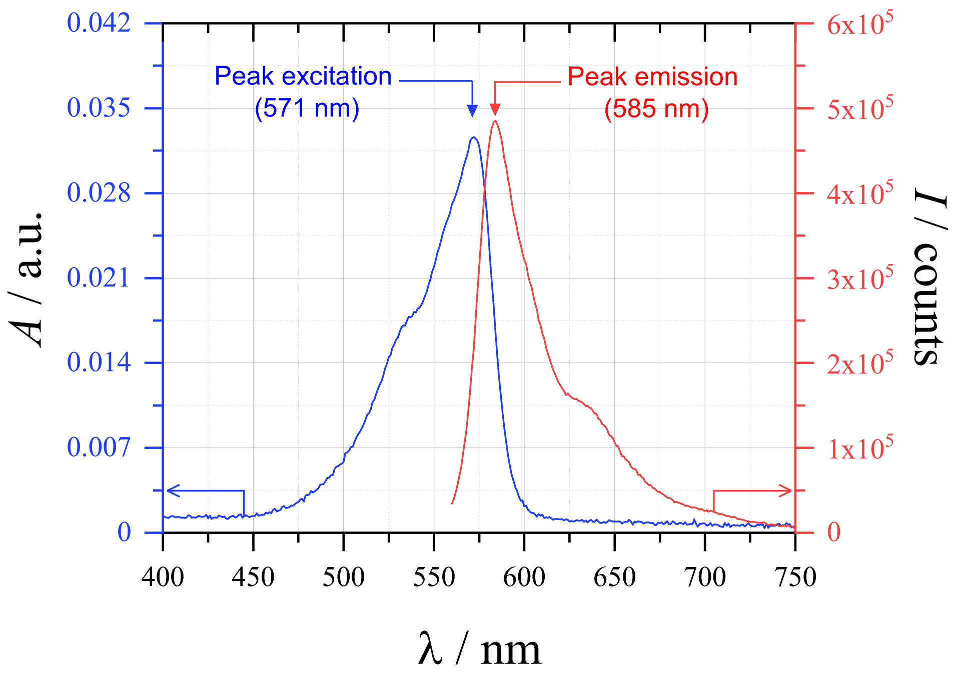

Figure S2: Absorbance (BLUE) and fluorescence (RED) spectra (excitation @ 550 nm) recorded using an aerobic solution of 0.5 M NaOH + 10 µM Amplex Red.

Figure S2 shows excitation and emission spectra recorded in a solution of Amplex Red in sodium hydroxide. In these conditions, the Amplex Red was rapidly photooxidised to resorufin, allowing the spectra to be recorded. The peak excitation and peak emission wavelengths were found to be in agreement with literature values of 571 and 585 nm respectively. It was determined from this that the TRITC-A filter set ($\lambda_{excitation}$ = 543–566 nm, $\lambda_{emission}$ = 582–636 nm) could be both used to sufficiently excite the resorufin and to measure the fluoresced light.

Influence and rate of photooxidation

The photooxidation of Amplex Red was demonstrated in a phosphate buffer saline solution. Fluorescence intensity profiles were recorded using a freshly made solution of Amplex Red in PBS, then the same intensity profiles were recorded after the solution had been left on a lab bench to photooxidise for two hours. In both cases, an electrode (either glassy carbon or carbon fibre) which was not connected to any potentiostat was held in the solution, and the microscope was focussed on the electrode surface to ensure that results were comparable.

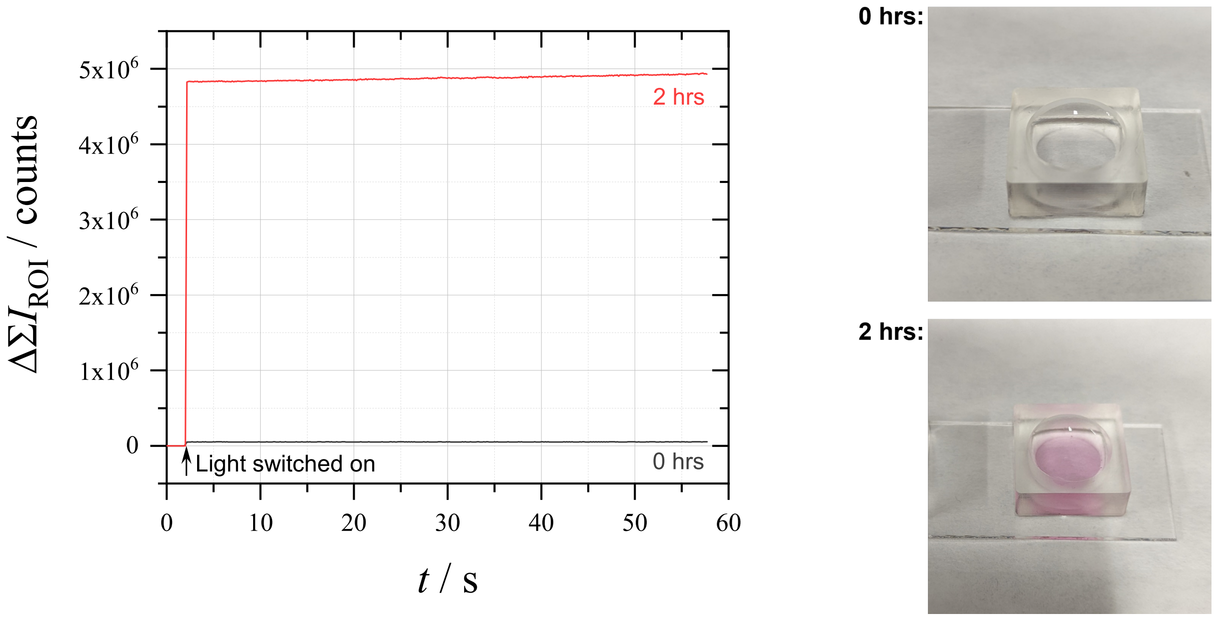

Figure S3: (LEFT) Change in sum fluorescence intensity measured through a 2.5× objective at an unconnected 3 mm Ø GC electrode in a (BLACK) freshly made and (RED) two hour old aerobic solution of 0.1 mM Amplex Red + 0.2 M PBS. (RIGHT) Images of a freshly made and two hour old aerobic solution of 0.1 mM Amplex Red + 0.2 M PBS.

Figure S3 shows the fluorescence intensity measured at a glassy carbon electrode through a 2.5× objective. No photooxidation is observed during the measurement, since the numerical aperture of the objective is quite low (0.075), which limits the collection of light. However, after the light was switched on, it is clear that the background intensity was much larger in the old solution. This suggests significant photooxidation had taken place, which can be confirmed by visual inspection of the solution in the accompanying images.

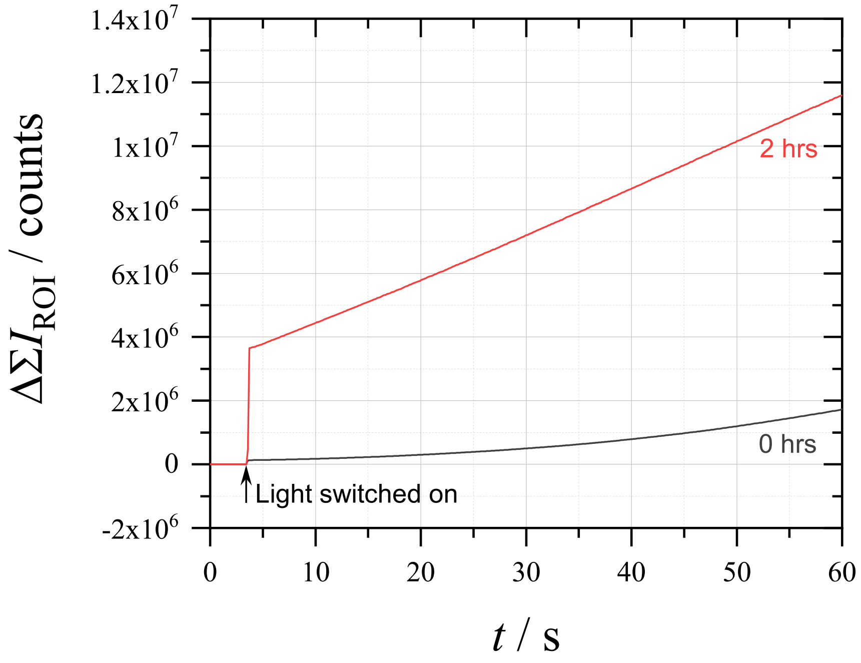

Figure S4: Change in sum fluorescence intensity measured through a 20× objective at an unconnected 33 µm Ø CF electrode in a (BLACK) freshly made and (RED) two hour old aerobic solution of 0.1 mM Amplex Red + 0.2 M PBS.

Figure S4 shows the fluorescence intensity measured at a carbon fibre electrode through a 20× objective. Due to the higher numerical aperture of this objective (0.5), the photooxidation is clearly visible by the shifting background intensity. It should also be noted that the amount of photooxidation during the measurement is much greater in the old solution.

Background electrochemistry of glucose on Pt and Au electrodes

Carbon electrodes were used to avoid the influence of glucose oxidation on the reporting electrode.

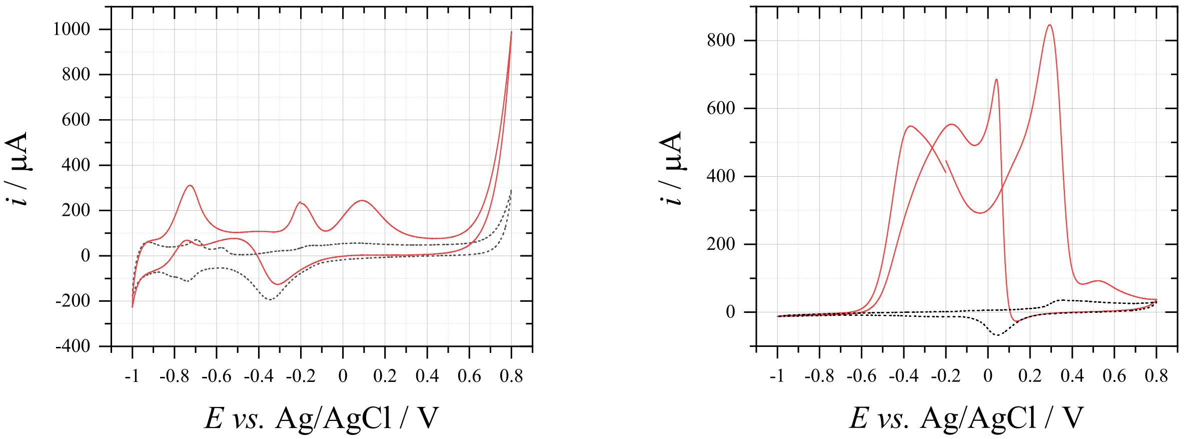

Figure S5: Cyclic voltammograms (500 mV s^{-1}) recorded on (LEFT) a 2 mm Ø Pt electrode and (RIGHT) a 2 mm Ø Au electrode in aerobic solutions of (BLACK, dashed) 0.5 M NaOH and (RED, solid) 0.5 M NaOH + 0.1 M $C_6H_{12}O_6$.

Figure S5 shows the significant activity of glucose observed on both Pt and Au electrodes, compared to the current observed in just sodium hydroxide. In the closed bipolar setup, some of the charge passed during ferrocenemethanol oxidation would be used to drive the oxidation of glucose on these materials, making them less appropriate as reporting electrodes.

Evidence for the passage of current when switching to OCP

The cell was kept on after most experiments, with the potential of the working electrode set to the value required for ferrocenemethanol reduction (0 V *vs.* Ag/AgCl). When switching to open circuit potential after an experiment, spikes in fluorescence intensity (as seen in Figure 4) were observed. This was investigated further using chronopotentiometry.

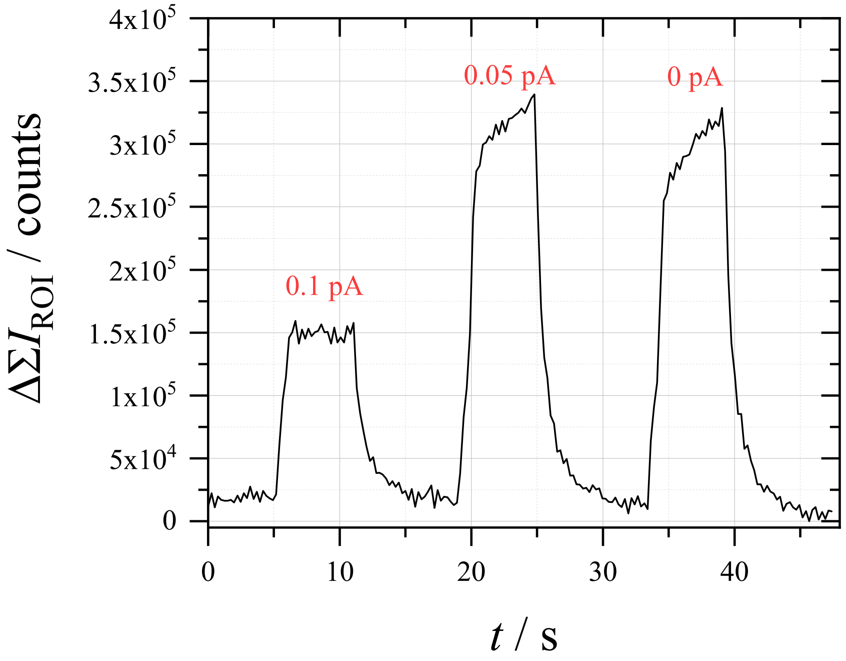

Figure S6: Change in sum fluorescence intensity measured at a 33 µm Ø CF electrode (reporting) in an aerobic solution of 10 µM Amplex Red + 0.1 M $C_6H_{12}O_6$ + 0.5 M NaOH whilst performing chronopotentiometry (*vs.* Ag/AgCl) to the indicated current levels on a 7 µm Ø CF electrode (detection) in an aerobic solution of 2.5 µM FcMeOH + 0.1 M K_2SO_4.

Figure S6 shows the fluorescence intensity profiles recorded during chronopotentiometry at extremely low currents. The intensity profile seen at 0.1 pA is similar to those seen at currents below 1 pA in Figure 9. The profile at 0.05 pA is closer to the profile observed when there is supposedly no current flowing. This indicates a limitation of the potentiostat in setting the current values, but more importantly suggests that there is still some current flowing at OCP. On this basis, it is theorised that switching to OCP can cause some temporary current flow which induces the fluorescence spikes observed experimentally.

Maximising the collection of fluorescence

Maximising the fluorescence signal naturally increases the sensitivity towards the detection of electrons. This can be achieved by increasing the incident light intensity and by using higher numerical apertures.

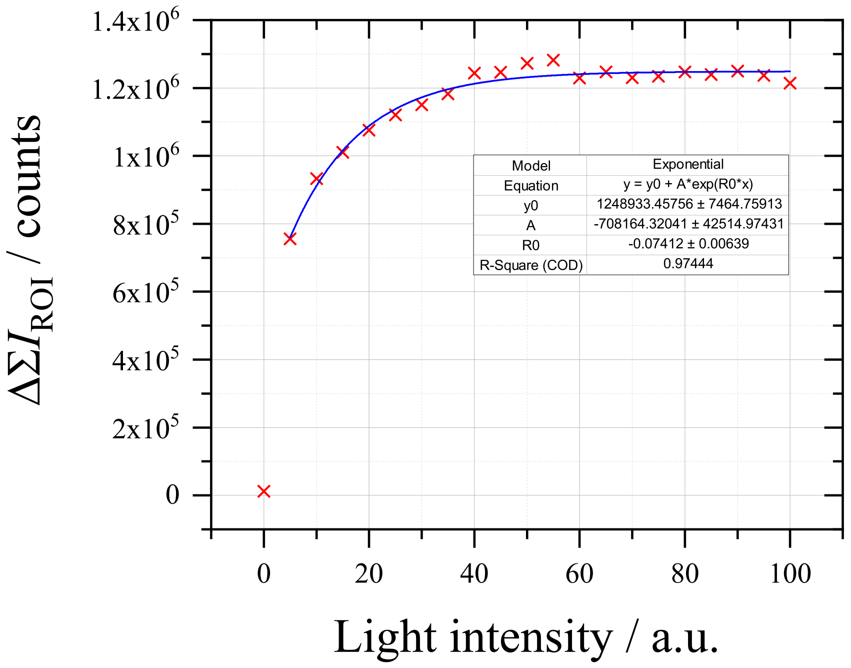

Figure S7: Change in sum fluorescence intensity vs. incident light intensity measured at a 33 µm Ø CF electrode (reporting) in an aerobic solution of 10 µM Amplex Red + 0.1 M $C_6H_{12}O_6$ + 0.5 M NaOH during continuous cyclic voltammetry (100 mV s^{-1}; *vs.* Ag/AgCl) measured at a 7 µm Ø CF electrode (detection) in an aerobic solution of 50 µM FcMeOH + 0.1 M K_2SO_4.

Figure S7 shows the change in fluorescence intensity measured during voltammetry using different incident light intensities. The fluorescence intensity reaches a maximum at around 50% of the maximum incident light intensity. Beyond this, the fluorescence intensity does not increase further, and may even start to experience photobleaching when the incident light is at full intensity. On this basis, the incident light was left at 50% for all experiments.

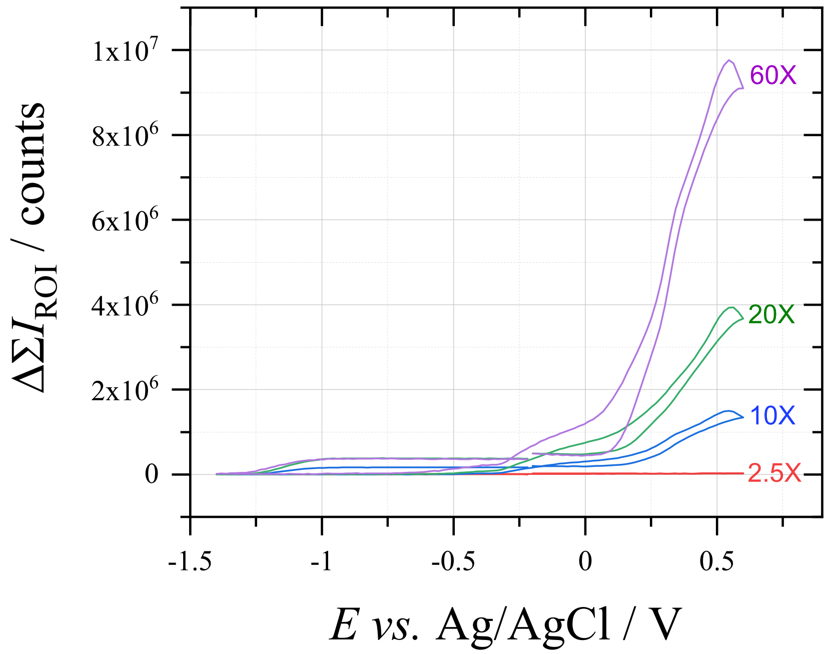

Figure S8: Change in sum fluorescence intensity measured during cyclic voltammetry ($100\,\mathrm{mV\,s^{-1}}$) at a 33 µm Ø CF electrode in an aerobic solution of 10 µM Amplex Red + 0.1 M $C_6H_{12}O_6$ + 0.5 M NaOH. The fluorescence was measured through (RED) a 2.5× objective, (BLUE) a 10× objective, (GREEN) a 20× objective, and (PURPLE) a 60× objective.

Figure S8 shows the fluorescence intensity measured through different microscope objectives during voltammetry of Amplex Red. Objectives with greater magnification also had higher numerical apertures, resulting in increased fluorescence intensities measured during the voltammetry.

Example calculation of the gradients from Figure 7

The gradient of integrated fluorescent count vs. charge passed has units of counts C^{-1}. This can be converted to photons C^{-1} using Equation 1, which was provided by Hamamatsu in the documentation alongside the CMOS camera.

$$\text{No. of photons} = \frac{CF \times \Delta I}{Q(\lambda)} \tag{1}$$

where CF is the conversion factor of the camera, ΔI is the measured intensity of pixels (background subtracted), and $Q(\lambda)$ is the quantum efficiency of the camera. This can be modified to give Equation 2

$$\text{Photons per electron} = \frac{CF}{Q(\lambda)} \times \frac{\Delta \int \Sigma I}{\Delta q} \times \frac{F}{N_A} \tag{2}$$

In this equation, the ΔI term is replaced with our gradient in counts C^{-1} and then multiplied by Faraday constant (F) divided by Avogadro's constant (N_A). We can use the values of $CF = 0.46$ photons count^{-1} and $Q(\lambda) = 0.8$ that are provided by Hamamatsu, and the value of our gradient.

$$\frac{0.46 \text{ photons count}^{-1}}{0.8} \times (4.98 \times 10^{15} \text{ counts C}^{-1}) \times \frac{96485 \text{ C mol}^{-1}}{6.022 \times 10^{23} \text{ mol}^{-1}} \tag{3}$$

$$= 0.000459 \text{ photons electron}^{-1}$$

By adding the error of the gradient and repeating the calculation, we can get the positive error of this photons per electron value.

$$\frac{0.46 \text{ photons count}^{-1}}{0.8} \times (5.28 \times 10^{15} \text{ counts C}^{-1}) \times \frac{96485 \text{ C mol}^{-1}}{6.022 \times 10^{23} \text{ mol}^{-1}} \tag{4}$$

$$= 0.000486 \text{ photons electron}^{-1}$$

By subtracting the error of the gradient and repeating the calculation, we can get the negative error of this photons per electron value.

$$\frac{0.46 \text{ photons count}^{-1}}{0.8} \times (4.69 \times 10^{15} \text{ counts C}^{-1}) \times \frac{96485 \text{ C mol}^{-1}}{6.022 \times 10^{23} \text{ mol}^{-1}} \tag{5}$$

$$= 0.000432 \text{ photons electron}^{-1}$$

Since both the positive and negative error are the same, the photons per electron value can be taken as 0.000459 ± 0.000027 photons electron^{-1}. The reciprocal of this value gives the electrons per photon.

$$= \frac{1}{0.000459 \text{ photons electron}^{-1}} \tag{6}$$

$$= 2178.5 \text{ electrons photon}^{-1}$$

With a positive error calculated using Equation 7.

$$= \frac{1}{0.000486 \text{ photons electron}^{-1}} \tag{7}$$

$$= 2316.5 \text{ electrons photon}^{-1}$$

And with a negative error calculated using Equation 8.

$$= \frac{1}{0.000432 \text{ photons electron}^{-1}} \tag{8}$$

$$= 2056.1 \text{ electrons photon}^{-1}$$

The positive error is larger, so we take our value as 2178.5 ± 138 electrons photon^{-1}.

Further evidence for the lingering response of the resorufin

Both the inverted hysteresis in the fluorescence intensity signal during voltammetry of Amplex Red (Figure 6) and the variance in electrons photon^{-1} during chronopotentiometry (Figure 8) indicated that the fluorophore lingers in solution after electrochemical generation.

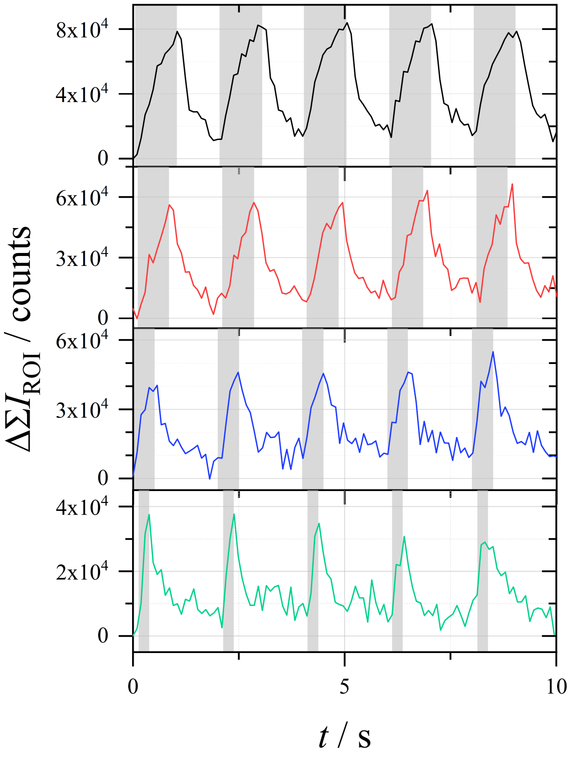

Figure S9: Change in sum of fluorescence intensity measured at a 33 µm Ø CF electrode (reporting) in an aerobic solution of 10 µM Amplex Red + 0.1 M $C_6H_{12}O_6$ + 0.5 M NaOH whilst performing a pulsed amperometric detection procedure (E dc = 0 V *vs.* Ag/AgCl; E pulse = 0.5 V *vs.* Ag/AgCl; interval time = 2 s; pulse time = 1 s (BLACK), 0.75 s (RED), 0.5 s (BLUE), and 0.25 s (GREEN)) on a 7 µm Ø CF electrode (detection) in an aerobic solution of 2.5 µM FcMeOH + 0.1 M K_2SO_4. The grey areas indicate when the working electrode was pulsed to an oxidation potential.

Figure S9 shows more evidence of this, measured during a pulsed amperommetric detection procedure. When the working electrode was pulsed to a potential for ferrocenemethanol oxidation (+0.5 V *vs.* Ag/AgCl), a peak in the fluorescence intensity was observed, but the width of this peak did not correlate to the duration of the pulse, suggesting the fluorophore lingers in solution.

Fluorescence signal in K_2SO_4 with and without FcMeOH

In Figure 8, a fluorescent signal was visible during the reporting of oxidation of diluted FcMeOH. Conversely, the expected current was below the capacitive current and was therefore not visible. To elucidate the different contributions of FcMeOH oxidation and double layer charging to the fluorescent signal, it was necessary to subtract the background signal recorded in only K_2SO_4.

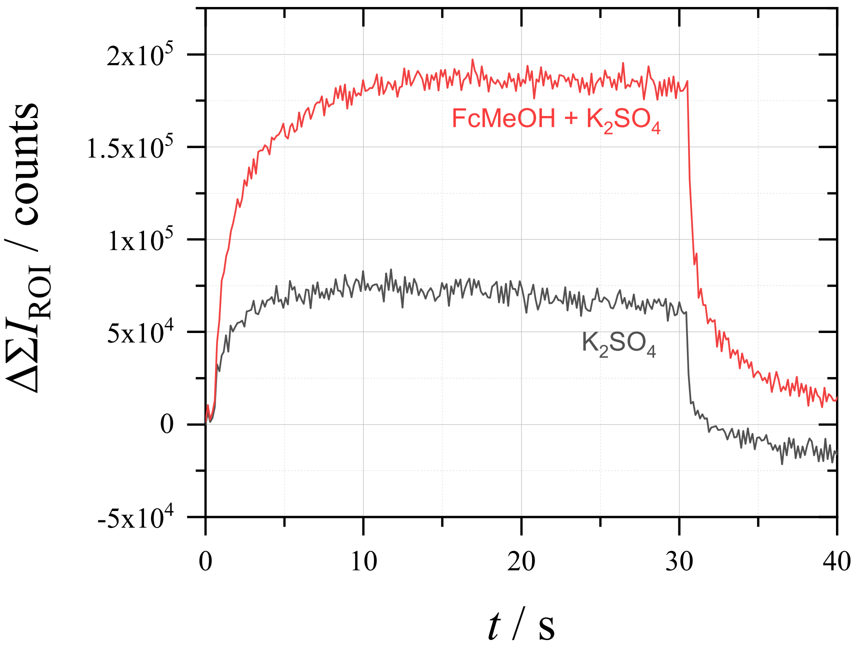

Figure S10: Change in sum fluorescence intensity measured at a 33 µm Ø CF electrode (reporting) in an aerobic solution of 10 µM Amplex Red + 0.1 M $C_6H_{12}O_6$ + 0.5 M NaOH during chronoamperometry (from 0 to +0.5 V *vs.* Ag/AgCl for 30 s) measured at a 7 µm Ø CF electrode (detection) in (BLACK) an aerobic solution of 0.1 M K_2SO_4 and (RED) an aerobic solution of 2.5 µM FcMeOH + 0.1 M K_2SO_4.

Figure S10 shows the difference between the fluorescent signals when chronoamperommetry of the working electrode was recorded with and without FcMeOH in the detection cell. Even without the FcMeOH, there was a noticeable fluorescence signal. This is due to Amplex Red oxidation caused by the passage of electrons from double layer charging, electromagnetic noise, and oxidation of trace impurities. These contributions are present in all experiments and can be reduced to some extent, but not fully removed. However, since single photons

can be resolved, it is unlikely that any background fluorescence will limit the capabilities of remote optical reporting to monitor electrochemistry below the detection limit.

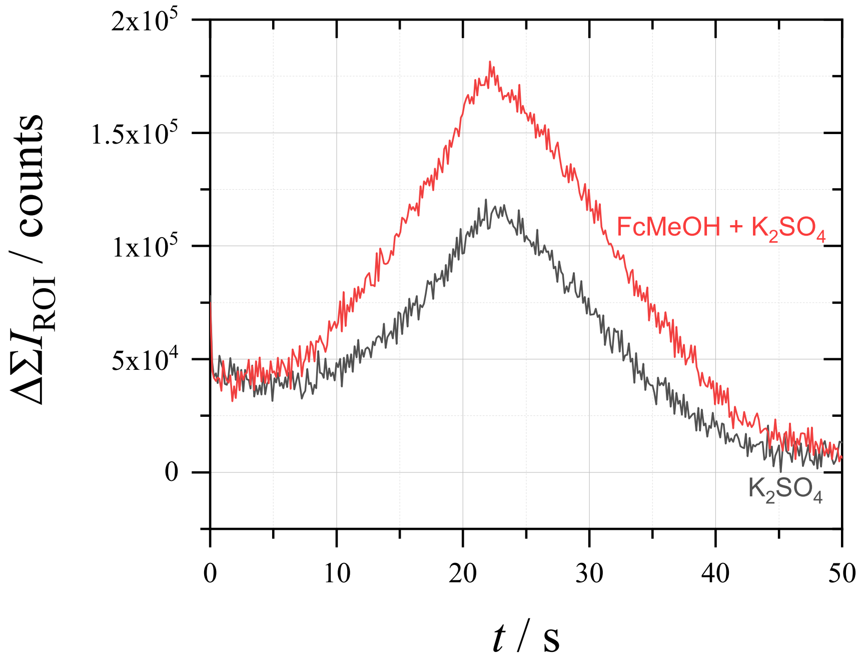

Figure S11: Change in sum fluorescence intensity measured at a 33 µm Ø CF electrode (reporting) in an aerobic solution of 10 µM Amplex Red + 0.1 M $C_6H_{12}O_6$ + 0.5 M NaOH during cyclic voltammetry (from 0 to +0.5 V *vs.* Ag/AgCl at 25 mV) measured at a 7 µm Ø CF electrode (detection) in (BLACK) an aerobic solution of 0.1 M K_2SO_4 and (RED) an aerobic solution of 2.5 µM FcMeOH + 0.1 M K_2SO_4.

Figure S11 shows the same relationship between a solution containing only K_2SO_4 and a solution containing both FcMeOH and K_2SO_4, except this time recorded with cyclic voltammetry. Once again the signal recorded in the presence of FcMeOH is greater than that recorded only in K_2SO_4 and the difference between the two signals could be used to extract some information on the amount of FcMeOH that was oxidised. It is also clear that the background fluorescence signal is driven by processes such as double layer charging and electromagnetic noise.

Example calculation of the integrated signal from Figures 7 and 10

In Figures 7 and 10, the chronopotentiometry at each current level was repeated 10 times. The fluorescence signals were integrated and averaged to provide each data point. An example of how this was done is shown using one of the chronopotentiometry experiments performed for Figure 7.

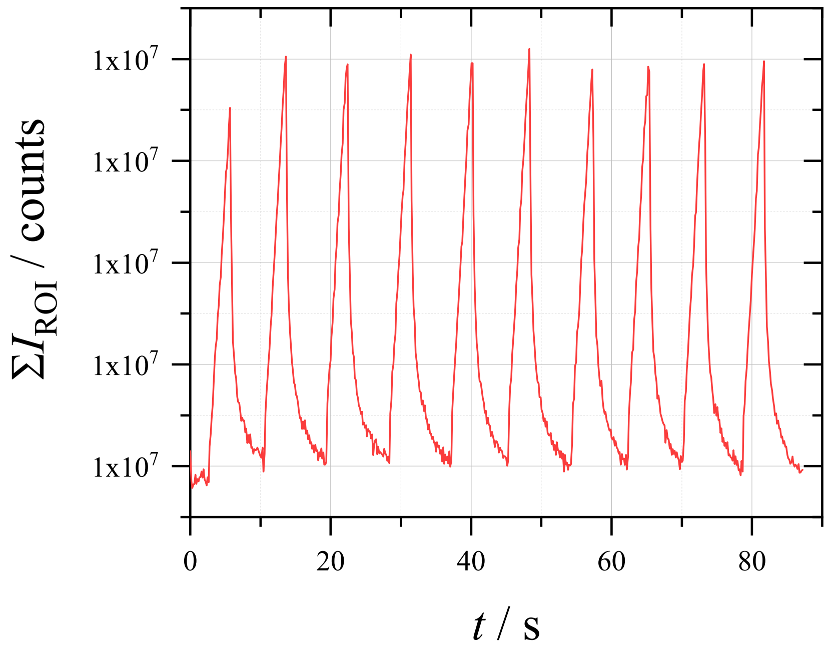

The fluorescence signal for 10 consecutive pulses can be seen. These are not background subtracted, since the integration can instead be done with a baseline. This is usually more accurate than subtracting the background, since in some longer experiments the background can shift over time.

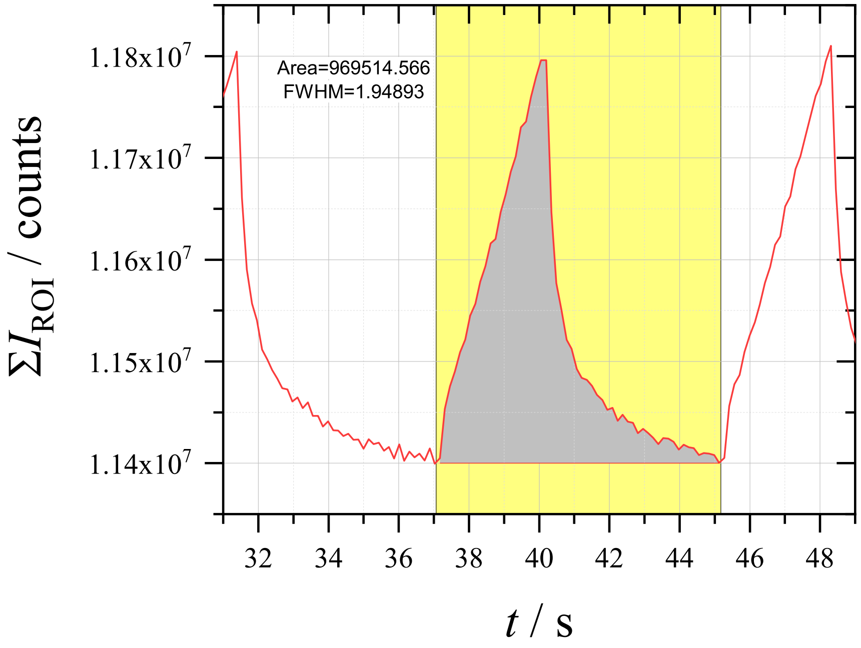

The pulse which is to be integrated is focussed on, and a baseline corresponding to the background before the pulse is applied. The integrated area underneath the pulse is used to calculate the average integrated change in the sum of fluorescence intensity.
